# Uterine leiomyoma, retained fetal cranial bones, and reproductive microbiome analysis in a fallow deer (*Dama dama*): a case report

**DOI:** 10.3389/fvets.2026.1872878

**Published:** 2026-06-23

**Authors:** Zsuzsanna Szőke, Péter Fehér, Szilamér Ferenczi, István Lakatos, Viktor Stéger, Ákos Sükösd, Farkas Sükösd, Levente Sára

**Affiliations:** 1 Reproductive Biology and Toxicology Group, Department of Animal Biotechnology, Institute of Genetics and Biotechnology, Hungarian University of Agriculture and Life Sciences, Godollo, Hungary; 2 Applied Wild and Farm Animal Genomics Group, Department of Genetics and Genomics, Institute of Genetics and Biotechnology, Hungarian University of Agriculture and Life Sciences, Godollo, Hungary; 3 Laboratory of Molecular Neuroendocrinology, Institute of Experimental Medicine, Hungarian Research Network, Budapest, Hungary; 4 Microbial Biotechnology and Microbiomics Group, Department of Microbiology and Applied Biotechnology, Institute of Genetics and Biotechnology, Hungarian University of Agriculture and Life Sciences, Godollo, Hungary; 5 Department of Regional Game Management, Ministry of Agriculture, Budapest, Hungary; 6 Department of Orthopedics, Semmelweis University, Budapest, Hungary; 7 Department of Pathology, Péterfy Sándor Street Hospital, Budapest, Hungary; 8 Institute of Biochemistry, Hungarian Research Network, Szeged, Hungary; 9 Department of Obstetrics and Gynecology, Semmelweis University, Budapest, Hungary

**Keywords:** case report, fallow deer, uterine leiomyoma, uterine/gut microbiome, wildlife

## Abstract

Pathological and microbiological surveillance of wildlife can reveal clinically silent but biologically important reproductive disorders. This case report describes a middle-aged (6–7 years) fallow deer hind (*Dama dama*) in good body condition, legally culled in Hungary, in which post-mortem examination identified a uterine leiomyoma in the left uterine horn and retained fetal cranial bones in the cranial vagina. To the best of our knowledge, this is the first published description of uterine leiomyoma in this species. Gross pathology, histopathology, and desmin immunohistochemistry supported the diagnosis of leiomyoma, and 16S rRNA amplicon sequencing was used to compare the microbiomes of the unaffected uterine horn, affected uterine tissue, and feces. The affected uterine sample showed a microbial profile more similar to that of feces than the unaffected uterine sample, with an increased relative abundance of genera, including *Bacteroides*, *Escherichia–Shigella*, and *Turicibacter*. As this was a single post-mortem case, no treatment was administered. These findings suggest a possible association between chronic mechanical obstruction, retained fetal material, and marked local microbial alteration, while also illustrating the limitations of causal inference from single-animal microbiome data. This case expands the differential diagnosis of reproductive tract lesions in wild ruminants and highlights the value of integrating pathology with careful microbiome interpretation in wildlife case reports.

## 1 Introduction

Documenting unusual pathological findings in free-ranging wildlife is scientifically valuable because such cases refine species-specific disease knowledge and provide comparative insights into wildlife management. Tumors of the female reproductive tract are considered rare in domestic ruminants, partly because these livestock animals often do not reach the age at which the risk of neoplasia increases significantly (1). Smooth muscle tumors, such as leiomyomas and leiomyosarcomas, account for only 1.00–2.00% of neoplastic diseases in cattle (1, 2). Leiomyomas are monoclonal benign tumors originating from the smooth muscle cells of the myometrium (1, 3). Although they are the most common uterine tumors in carnivores, such as elderly dogs (4), they are rarely diagnosed in domestic ruminants (3). The exact etiology of their development is not fully understood; however, the literature agrees that they are steroid hormone-dependent lesions, particularly responsive to estrogens (1, 3).

These tumors mostly grow slowly and can remain clinically asymptomatic for a long time (3). However, if their size causes them to occupy a significant portion of the uterine wall or lumen, they can severely reduce reproductive efficiency by forming mechanical barriers (1, 3). Based on documented cattle cases, leiomyomas can cause infertility, abortion, or dystocia by obstructing the birth canal at the end of gestation (3). Occasionally, tumors inhibit prostaglandin (PGF2α) secretion in the inner layer of the uterus, leading to the persistence of the corpus luteum and a prolonged absence of estrus (1). Uterine leiomyomas have also been documented in sheep (5); however, their occurrence and population-level impact on wild ruminants, including fallow deer, remain virtually unexplored. Based on the current literature, this case represents the first documented occurrence of a uterine leiomyoma in a fallow deer.

This case report details post-mortem findings in a middle-aged fallow deer hind legally culled during population management in Hungary. The coexistence of a uterine smooth muscle tumor and retained fetal cranial bones prompted a complementary microbiome analysis of unaffected uterine tissue, affected uterine tissue, and feces. The aim was not to establish causation but to characterize the pathological findings and assess whether the affected uterine microenvironment showed evidence of microbial alteration relative to the contralateral horn and fecal reference material.

## 2 Case description

To ensure accuracy and transparency, this case report was prepared in compliance with the CARE guidelines. The completed CARE checklist is available in Supplementary Table S1.

### 2.1 Necropsy and sample collection

**Figure 1:**
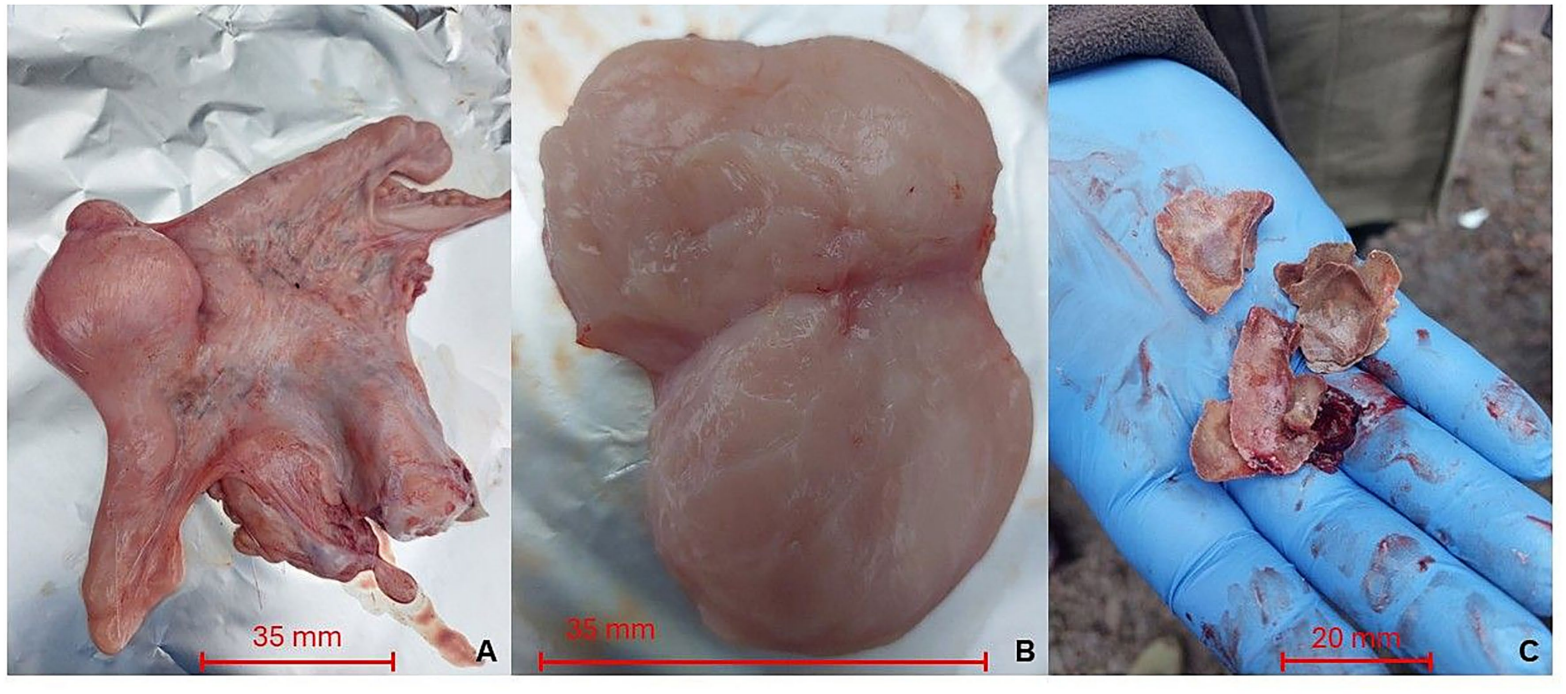
Macroscopic findings in the reproductive tract of fallow deer (*Dama dama*). **(A)** Excised reproductive tract showing a prominent mass (leiomyoma) located in the left uterine horn. **(B)** Macroscopic appearance of a benign smooth muscle tumor. **(C)** Retained fetal cranial bone fragments from a previous pregnancy were recovered from the upper part of the vagina.

Following evisceration, the body condition score (BCS) of the fallow deer was determined by assessing the kidney fat index. The specimen under examination was a 6- to 7-year-old female fallow deer (6) with a carcass weight of 38 kg (excluding head, distal limbs and viscera). Ovarian examination confirmed the presence of a *corpus luteum*, indicative of an active reproductive cycle; however, the animal was neither pregnant nor lactating at the time of culling. Furthermore, no macroscopic systemic lesions were observed, and the overall body condition score (BCS) was assessed as adequate (7). Subsequently, the reproductive tract (uterus, ovaries, and vagina) was dissected for evaluation and sampling. The inflamed tissue measured 30 × 35 mm. Originating from the myometrium on the inner-upper aspect of the left uterine horn, the mass was covered by the *tunica serosa*. Due to its size, it presented as a subserosal and partially intramural lesion, positioned 1 cm laterally from the intercornual ligament. It partially extended into and deformed the uterine cavity and was located 4–5 cm away from the left ovary. To obtain reference samples for the gut microbiome, the large intestine was collected using a sterile scalpel to collect feces. Strict aseptic techniques were maintained throughout the procedure using sterile gloves and storage containers to minimize the risk of cross-contamination. Immediately after collection, the uterine and fecal samples were placed on dry ice for preservation. Upon arrival at the laboratory, all samples were stored continuously at −70 °C until microbial DNA extraction (7) was initiated (Figure 1).

### 2.2 Timeline

A conventional clinical history was unavailable because this was a free-ranging wildlife case identified post-mortem. However, the pathological findings and subsequent laboratory procedures support the following chronology: (i) a complicated pregnancy resulted in fetal demise and the retention of cranial bones in the cranial vagina; (ii) the retained fetal material and concurrent uterine obstruction by a leiomyoma were accompanied by local inflammation and microbial colonization; (iii) the lesions were identified at necropsy after legal culling on December 6, 2025; and (iv) between January and February 2026, DNA extraction, 16S rRNA amplicon sequencing, and bioinformatic analyses were conducted to evaluate the microbiome profile of the lesions. The exact timing of fetal death and the duration of retention could not be determined from the available material.

### 2.3 Pathological examination

Following evisceration and body condition assessment, the reproductive tract (uterus, ovaries, and vagina) was dissected. The aim of the macroscopic pathological examination was to identify and describe any visible pathological changes.

Formalin-fixed, paraffin-embedded tissue samples were sectioned at 4 μm thickness and stained with hematoxylin and eosin (H&E) according to standard protocols for routine histopathological examination. Immunohistochemical staining was performed on 4-μm-thick sections using the Ventana BenchMark Ultra automated staining platform (Roche/Ventana). Following deparaffinization, antigen retrieval, and incubation with the Desmin anti-human antibody (Ventana Roche, Clone DE-R-11, mouse mAb), the procedure was carried out according to the manufacturer’s protocol. Detection was performed using the UltraView detection system, followed by hematoxylin counterstaining (8).

**Figure 2:**
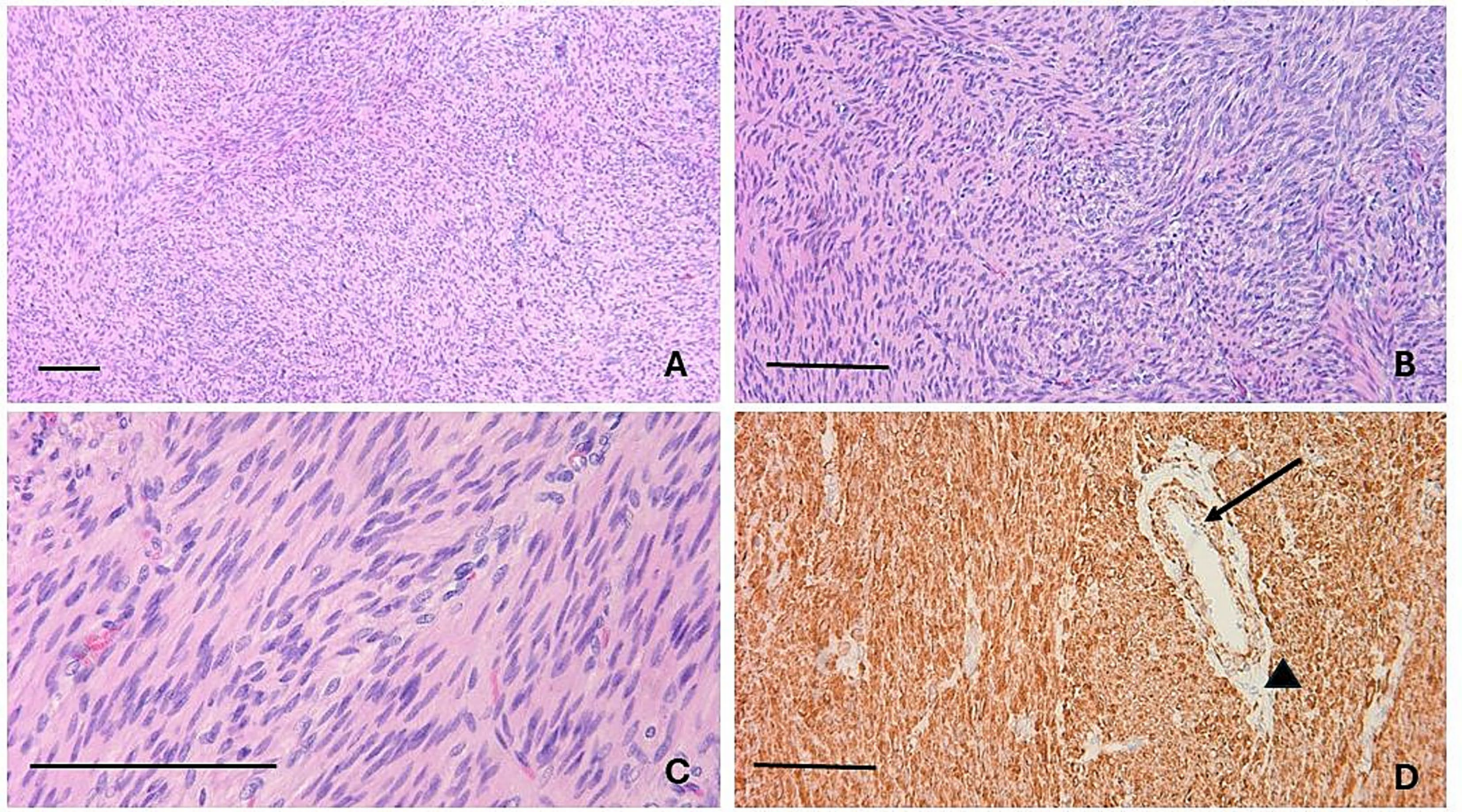
The histological and immunohistochemical (IHC) findings are consistent with a leiomyoma. **(A)** The benign mesenchymal tumor shows a fascicular growth pattern (H&E, 5×). **(B)** The uniform spindle cells have eosinophilic cytoplasm and elongated, blunt-ended (“cigar-shaped”) nuclei (H&E, 10×). **(C)** No significant cytologic atypia is identified. Mitotic activity is low, and tumor cell necrosis is absent (H&E, 20×). **(D)** Immunohistochemical staining with smooth muscle-specific Desmin antibody shows strong cytoplasmic expression in the tumor cells. The muscular layer of the small vein in the center is positive (arrow), while its unstained adventitia serves as an internal negative (arrowhead) control (Desmin IHC 10×). Scale bar is equal to 100 μm.

Histological examination was performed on a fragmented, firm tissue specimen detached from the serosal surface of the uterine fundus, measuring approximately 4 cm in greatest diameter. In the section, the lesion showed a grey-white, whorled appearance. The relationship of the lesion to the surrounding tissue could not be assessed due to specimen fragmentation. Microscopic and immunohistochemical examination (IHC) (Figure 2) was consistent with a benign mesenchymal tumor, namely, a leiomyoma. A fascicular growth pattern composed of uniform spindle cells with eosinophilic cytoplasm and elongated, blunt-ended (“cigar-shaped”) nuclei was observed. No significant cytological atypia was identified. Mitotic activity was low, and tumor cell necrosis was absent. Immunohistochemical staining with the human smooth muscle-specific Desmin antibody showed strong cytoplasmic expression in the tumor cells. Positive staining of the muscular layer of the small vein in the center served as a positive control, and the unstained adventitia served as an internal negative control. The IHC supported the smooth muscle origin of the tumor.

### 2.4 Sampling and DNA extraction

Genomic DNA was extracted from approximately 200 mg of starting material for each sample using the MagCore Gut Microbiome DNA Kit Standard Protocol (RBC Bioscience, Taiwan) on a MagCore Automated Nucleic Acid Extractor, following the manufacturer’s instructions. The quantity of extracted DNA was measured using a Qubit Flex Fluorometer with the Qubit 1X dsDNA HS Assay Kit (Thermo Fisher Scientific, United States), and DNA quality and integrity were assessed using an Agilent 4150 TapeStation system with a D5000 ScreenTape assay (Agilent Technologies, United States).

The following three samples were subjected to microbiome analysis:

7454/1: Visibly normal endometrium (from visibly normal uterine horn).

7454/2: Inflamed tissue (from the tumorous uterine horn).

7454/3: Feces/colon sample (reference from the gastrointestinal tract).

### 2.5 Microbiome analysis (16S rRNA)

To identify the bacterial communities, we performed Illumina 16S rRNA amplicon sequencing (with an average quality of > Q30). To target the V3-V4 hypervariable regions of the 16S rRNA gene, we utilized a highly evaluated bacterial primer pair previously described by (9). These gene-specific sequences were modified by incorporating the Illumina adapter overhangs. The complete primer sequences, represented using the standard International Union of Pure and Applied Chemistry (IUPAC) nucleotide nomenclature (where N = any base; W = A or T; H = A or C or T; and V = G or C or A), were as follows:

16S Amplicon PCR Forward Primer = 5′.

TCGTCGGCAGCGTCAGATGTGTATAAGAGACAGCCTACGGGNGGCWGCAG.

16S Amplicon PCR Reverse Primer = 5′.

GTCTCGTGGGCTCGGAGATGTGTATAAGAGACAGGACTACHVGGGTATCTAATCC.

For PCR amplification, 1 μL of the extracted DNA template was used as input, and the PCR program consisted of 30 amplification cycles. To monitor assay performance and ensure the absence of background contamination, both positive control and negative (no-template) controls were included during the PCR runs. Following amplification, sequencing libraries were indexed using Illumina CD96 indexes. Sequencing was performed on an Illumina MiSeq platform using the MiSeq Reagent Kit v2 (500-cycle), generating 2 × 250 bp paired-end reads.

### 2.6 Bioinformatic analysis

Amplicon-based metagenomic data analysis was performed using the MyDADA2 workflow (version 1.31). Initial primer trimming was conducted using Cutadapt (version 4.4). During this trimming process, a minimum read length of 50 nt was enforced, zero ambiguous (“N”) nucleotides were permitted in the input reads, and untrimmed reads were discarded.

Subsequent read processing, denoising, and abundance analyses were performed using the DADA2 package (version 1.36.0) in R. The reads were not subjected to quality value (QV)-based trimming or fixed-length truncation at the ends. The filtering parameters strictly allowed zero “N” calls and a maximum of two expected errors (maxEE) for both forward and reverse reads. Reads shorter than 100 nt were removed. Forward and reverse reads were merged, requiring a minimum overlap of 12 nt with zero mismatches allowed in the overlapping region. The resulting Amplicon Sequence Variants (ASVs) were length-filtered to retain sequences between 350 and 500 nt. Chimeric sequences were identified and removed from the dataset. For samples with low biomass (such as the uterus), a relative frequency threshold of 0.1% is commonly used to avoid contamination (e.g., (10)); therefore, we also used this threshold in this case. Accordingly, we applied this threshold to our dataset and classified ASVs with a total read count of 65 or fewer as background noise and excluded them from further analysis.

Taxonomic assignment of the filtered ASVs was performed using the Silva 16S NR99 v138.2 reference sequence database, with a minimum bootstrap confidence threshold of 75, and both read orientations were used to identify the best hit. Finally, to assess sampling depth and diversity, rarefaction curves were generated, and alpha diversity indices (including Chao1 and Shannon) were calculated. Taxonomic composition was visualized by assessing the abundance of the top 20 taxa across different taxonomic ranks (e.g., at the genus level).

Following sequencing and initial quality control, the amplicon reads from the three analyzed samples were processed using the DADA2 workflow. Across the dataset, the denoising and merging processes identified 2,227 unique Amplicon Sequence Variants (ASVs). Strict filtering parameters were applied to ensure data integrity; consequently, 1,206 sequences were identified as chimeras and were subsequently removed. Additionally, eight ASVs were excluded from the downstream analysis because their lengths fell outside the predefined threshold of 350 to 500 nucleotides (Supplementary Figure S1).

Analysis of the alpha diversity indicators revealed that the inflamed tumorous tissue exhibited the highest species richness among the sampled sites (Table 1).

**Table 1:** Alpha diversity indicators (Shannon, Chao1, and Simpson indices) in the samples studied.

Sample name	Sample type	Shannon index	Chao1 index	Simpson index
7454/1: Visibly normal endometrium (from visibly normal uterine horn)	Visibly normal endometrium	5.60	410	0.9948
7454/2: Inflamed tissue (from the tumorous uterine horn)	Tumorous/inflamed	5.65	410	0.9941
7454/3: Feces/colon sample (reference from the gastrointestinal tract)	Feces (colon)	5.43	340	0.9935

**Figure 3:**
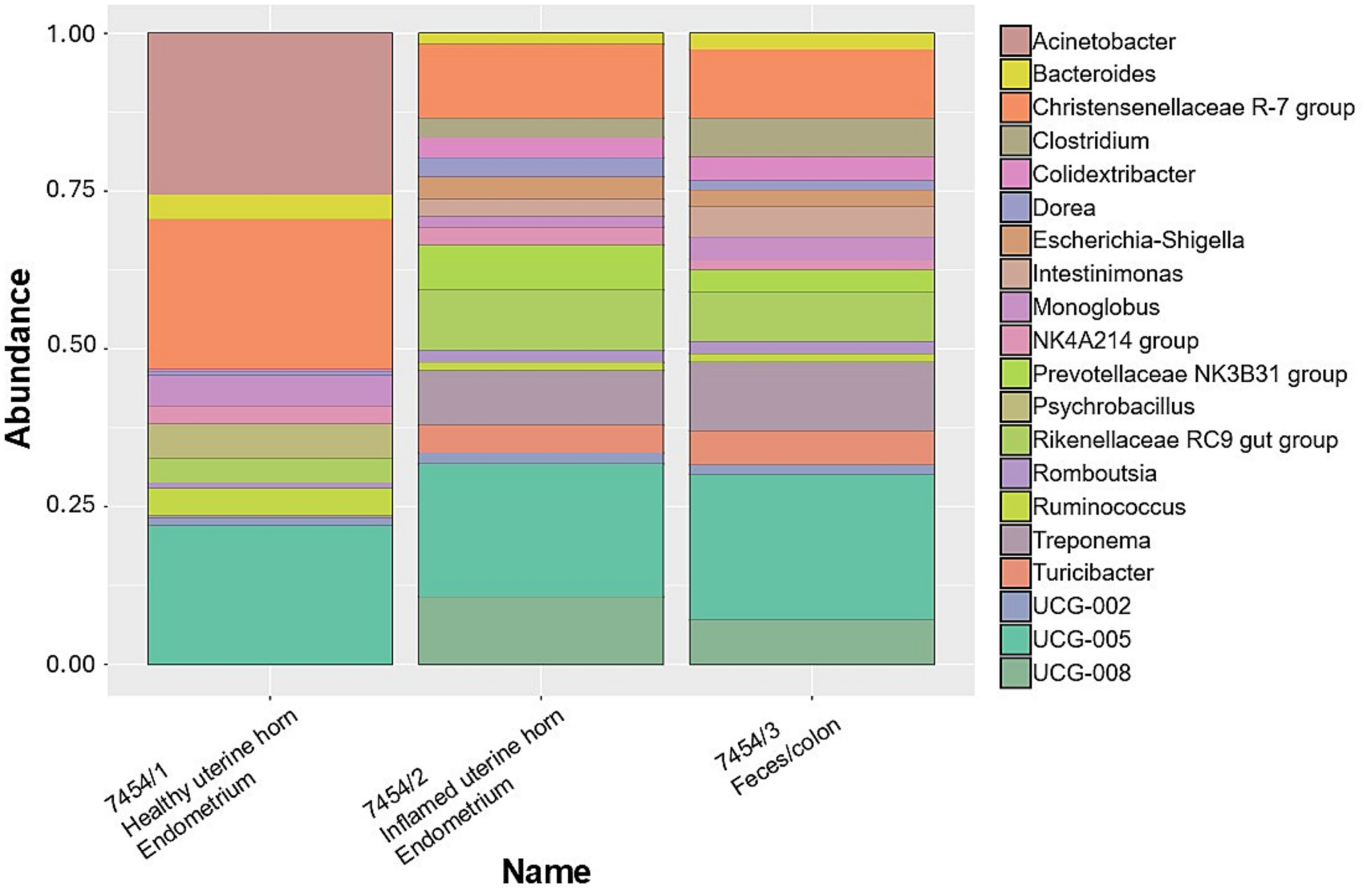
Relative abundance of the top 20 bacterial genera across sampled sites. The stacked bar chart displays the taxonomic composition of the microbiome in the visibly normal endometrium (7454/1), tumorous/inflamed tissue (7454/2), and fecal control samples (7454/3). Inflamed uterine tissue demonstrates profound dysbiosis, characterized by a drastic shift toward a fecal-like profile dominated by anaerobic and opportunistic pathogens.

Taxonomic profiling demonstrated distinct microbial compositions between the different tissue states. Several differences were observed between the microbiome profiles of the inflamed uterine tissue and those of visibly normal endometrial tissue. Specifically, the tumorous tissue was characterized by a high relative abundance of taxa that are frequently associated with gastrointestinal and anaerobic environments, including *Bacteroides*, *Escherichia–Shigella*, *Romboutsia, Turicibacter*, and representatives of the *Oscillospiraceae* (UCG groups) and *Rikenellaceae* families (Figure 3).

Several genera, such as the *Prevotellaceae* NK3B31 group (932 reads), *Turicibacter* (578 reads), *Clostridium* (409 reads), *Intestinimonas* (363 reads), and *Escherichia-Shigella* (466 reads), were detected in the inflamed uterine tissue, but were absent in the visibly normal endometrium. Conversely, the genera *Acinetobacter* (2,343 reads) and *Psychrobacillus* (502 reads) were found exclusively in the visibly normal endometrial tissue. The presence of *Brucella* species was not confirmed in any sample (0 reads). The genus *Mycobacterium* was not detected above the applied relative frequency threshold in this dataset.

## 3 Discussion

An integrated assessment of pathological and metagenomic data offers distinctive insights into the reproductive biology of wild ruminants.

### 3.1 Evaluation of uterine leiomyoma

While leiomyomas are common in domestic animals (4, 11), their occurrence in wild animals is rare. *Rhabdomyosarcoma* has been reported in fallow deer (12), whereas leiomyomas have been documented in endangered species, such as the Sumatran rhinoceros (13). The current case contributes valuable information to the reproductive oncology of fallow deer. Macroscopically, the cut surface of the tumor appeared clean, with no evidence of hemorrhage. The absence of hemorrhage or colliquative necrosis suggests that the tumor maintained an adequate blood supply and did not experience ischemia, a common cause of secondary bleeding in large myomas. Moreover, the complete lack of macroscopic signs of inflammation or infection within the solid tumor tissue made an abscess unlikely. The well-circumscribed structural boundaries of the lesion are consistent with the typical morphological features of a benign neoplasm, such as a leiomyoma.

### 3.2 Chronic inflammation and fecal microbiome shift

As estimated in the timeline, the prolonged persistence of fetal bones for approximately 10 months may have resulted from mechanical (fetopelvic disproportion) or infectious factors (14). Notably, *Brucella* spp. were completely undetected (0 reads) in our metagenomic dataset. Species within the *Brucella* genus are well-recognized pathogens responsible for infectious abortion in domestic and wild ruminants. The lack of *Brucella* sequences in the 16S rRNA profile suggests that it may not have been the primary infectious driver of the initial miscarriage, indicating that the handling of the carcass, reproductive tract, and macerated fetal tissues likely did not pose an immediate zoonotic transmission risk to the hunters, wildlife managers, and veterinarians involved (15, 16). However, definitive exclusion of brucellosis would require targeted official diagnostic methods, such as specific qPCR or bacterial culture.

Similarly, the differential diagnosis considered *Mycobacterium bovis* infection. The macroscopic appearance of the tumor lacked caseous necrosis or typical tubercles. Furthermore, although the opportunistic pathogen *Mycobacterium* was detected in the inflamed tissue, its relative abundance was extremely low (only 15 reads). While these combined macroscopic and metagenomic findings do not support a classic bovine tuberculosis infection, specific diagnostic tests would be necessary to definitively exclude its presence (17).

After abortion, these retained bony structures function as foreign bodies and gateways for ascending infections (18). Microbiome analysis revealed a complex microbial profile within the tumor tissue. In contrast to acute pyometra, which is associated with reduced diversity, highly diverse polymicrobial colonization was observed. Approximately 80–90% of the flora consisted of typical gut bacteria (e.g., *Acetitomaculum*, *Phascolarctobacterium*, and *Alloprevotella*). The surface of the tumor provides an anaerobic environment and biofilm conducive to severe inflammation (19). *E. coli* facilitates the colonization of anaerobic abscess-forming bacteria (e.g., *Lawsonella* and *Solobacterium*), and the presence of *Treponema* contributes to further tissue destruction. The appearance of *Bibersteinia* demonstrates how opportunistic bacteria can become pathogenic under chronic stress. Despite this extensive presence of bacteria, the animal did not develop septic shock, suggesting local encapsulation of the infection.

### 3.3 Uterine dysbiosis and comparative mucosal pathology

The differences in microbiota composition between inflamed and visibly normal uterine horns revealed significant dysbiosis, extending beyond mere local infection. In the visibly normal endometrium, the exclusive presence of *Acinetobacter* and *Psychrobacillus* highlights distinct microbial communities that may contribute to or signify an undisturbed environment. Although *Acinetobacter* can function as an opportunistic pathogen, its increased abundance has also been linked to healthy mucosal surfaces in other comparative models, illustrating how certain genera may be indicative of visibly normal tissue (20).

In contrast, the complete replacement of the baseline flora by genera such as the *Prevotellaceae* NK3B31 group, *Turicibacter*, *Clostridium*, and *Escherichia-Shigella* in inflamed tissue indicates a marked deviation from physiological norms. This pattern is strongly consistent with broader observations of uterine dysbiosis, which is typically characterized by an increased prevalence of *Proteobacteria* (e.g., *Escherichia-Shigella*) and *Bacteroidetes* (e.g., *Prevotellaceae*) and the depletion of normal flora (21).

Moreover, these findings align with the dysbiosis hypothesis described for other mucosal tissues. The observed dynamics in this tumorous uterus, where inflammation is associated with a significant alteration in the microbial community structure, characterized by a reduction in potential commensals and an increase in opportunistic pathobionts, reflect microbiota changes documented in chronic inflammatory conditions such as inflammatory bowel disease (22, 23).

Identifying specific bacterial shifts, such as the emergence of *Prevotellaceae* and *Escherichia-Shigella* alongside the disappearance of *Acinetobacter*, not only corroborates the role of microbial dysbiosis in wildlife uterine pathology but also underscores taxa that could potentially serve as biomarkers for this condition. In future veterinary and wildlife management applications, monitoring the presence or absence of these key genera could facilitate the development of novel diagnostic tools for assessing the reproductive health of wild ruminant populations.

### 3.4 Limitations

Several limitations of this study must be acknowledged. First, this was a single-case report without population-based controls or biological replicates. Consequently, the findings represent a singular pathological event and cannot be generalized to the broader population of fallow deer. Second, because the subject was a free-ranging wild animal identified post-mortem, a comprehensive ante-mortem clinical history was unavailable, preventing a precise temporal correlation between the development of the uterine leiomyoma, fetal demise, and microbial shifts. Third, although sampling was conducted using strict aseptic techniques in the field, the possibility of environmental or post-mortem contamination cannot be entirely excluded. Fourth, the uterus is inherently a low-biomass microbial environment. This low bacterial biomass poses inherent challenges for 16S rRNA amplicon sequencing, necessitating the cautious interpretation of low-abundance taxa. Finally, although the 16S rRNA amplicon sequencing utilized in this study provides a comprehensive overview of the microbial community composition, it cannot definitively exclude the presence of specific zoonotic pathogens—such as *Brucella* and *Mycobacterium* species—with absolute certainty. An optimal approach to fully substantiate these conclusions would involve the combined interpretation of results from multiple methodologies. Reliable identification or exclusion of these specific pathogens would require targeted bacteriological culture or specific molecular diagnostic assays, such as conventional PCR, quantitative real-time PCR (qPCR), or loop-mediated isothermal amplification (LAMP).

## 4 Conclusion

This case report documents the concurrent presence of a uterine leiomyoma and retained fetal cranial bones in a middle-aged (6–7 years) fallow deer hind, along with descriptive differences in microbial composition between unaffected and affected uterine tissues, and feces. These findings support the interpretation that chronic reproductive tract obstruction and retained fetal material may coexist with marked local microbial alterations in free-ranging wildlife. More broadly, this case highlights the value of combining pathology and carefully interpreted microbiome data when investigating subclinical reproductive lesions in wild ruminants.

## Data Availability

The datasets presented in this study are available in online repositories. The names of the repository/repositories and accession number(s) are as follows: NCBI BioProject PRJNA1446681, with associated BioSample accessions SAMN56818641, SAMN56818642, and SAMN56818643.

## Supplementary material

Supplementary Figure 1 Rarefaction curves of 16S rRNA gene sequencing data. The graph illustrates the sequencing depth and estimated species richness (alpha diversity) across the three analyzed samples: normal endometrium (blue), tumorous/inflamed tissue (red), and feces (green). The curves plateau, indicating that the sequencing depth was sufficient to capture the vast majority of the microbial diversity present in each sample. Notably, inflamed tissue exhibited the highest species richness, in contrast to typical acute infections, suggesting diverse polymicrobial colonization.
