# Supplementary figures and images for: Uterine leiomyoma, retained fetal cranial bones, and reproductive microbiome analysis in a fallow deer (Dama dama): a case report
Source: Front Vet Sci. 2026 Jun 23;13:1872878. doi: 10.3389/fvets.2026.1872878 (PMC13337424; doi:10.3389/fvets.2026.1872878)

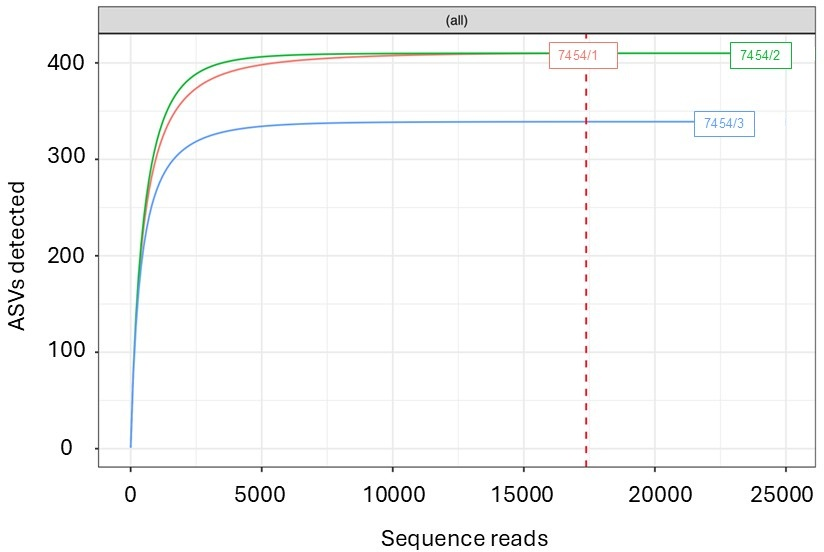

Supplement: Supplementary Figure 1 — Rarefaction curves of 16S rRNA gene sequencing data. The graph illustrates the sequencing depth and estimated species richness (alpha diversity) across the three analyzed samples: normal endometrium (blue), tumorous/inflamed tissue (red), and feces (green). The curves plateau, indicating that the sequencing depth was sufficient to capture the vast majority of the microbial diversity present in each sample. Notably, inflamed tissue exhibited the highest species richness, in contrast to typical acute infections, suggesting diverse polymicrobial colonization. [file Image_1.tif]
